# Shortwave infrared hyperspectral imaging as a novel method to elucidate multi-phase dolomitization, recrystallization, and cementation in carbonate sedimentary rocks

**DOI:** 10.1038/s41598-021-01118-4

**Published:** 2021-11-05

**Authors:** Cole A. McCormick, Hilary Corlett, Jack Stacey, Cathy Hollis, Jilu Feng, Benoit Rivard, Jenny E. Omma

**Affiliations:** 1 Department of Earth and Environmental Sciences, The University of Manchester, Manchester, M13 9PL UK; 2 Department of Physical Sciences, MacEwan University, Edmonton, AB T5J 4S2 Canada; 3 Department of Earth and Atmospheric Sciences, University of Alberta, Edmonton, AB T6G 2E3 Canada; 4 Rocktype Ltd., Magdalen Centre, Robert Robinson Avenue, Oxford, OX4 4GA UK

**Keywords:** Solid Earth sciences, Geochemistry, Mineralogy, Sedimentology

## Abstract

Carbonate rocks undergo low-temperature, post-depositional changes, including mineral precipitation, dissolution, or recrystallisation (diagenesis). Unravelling the sequence of these events is time-consuming, expensive, and relies on destructive analytical techniques, yet such characterization is essential to understand their post-depositional history for mineral and energy exploitation and carbon storage. Conversely, hyperspectral imaging offers a rapid, non-destructive method to determine mineralogy, while also providing compositional and textural information. It is commonly employed to differentiate lithology, but it has never been used to discern complex diagenetic phases in a largely monomineralic succession. Using spatial-spectral endmember extraction, we explore the efficacy and limitations of hyperspectral imaging to elucidate multi-phase dolomitization and cementation in the Cathedral Formation (Western Canadian Sedimentary Basin). Spectral endmembers include limestone, two replacement dolomite phases, and three saddle dolomite phases. Endmember distributions were mapped using Spectral Angle Mapper, then sampled and analyzed to investigate the controls on their spectral signatures. The absorption-band position of each phase reveals changes in %Ca (molar Ca/(Ca + Mg)) and trace element substitution, whereas the spectral contrast correlates with texture. The ensuing mineral distribution maps provide meter-scale spatial information on the diagenetic history of the succession that can be used independently and to design a rigorous sampling protocol.

## Introduction

Hyperspectral imaging involves the collection and analysis of reflectance data in the form of many, narrow and contiguous, spectral bands^1,2^. Laboratory-, field-, and satellite-based spectroscopy are well-established methods with numerous geological applications, including lithological mapping^3,4^, mineral prospectivity^5–7^, and environmental monitoring^8,9^. The application of such methods to carbonate rocks, however, is limited, with previous research largely focusing on the measurement of the laboratory-based spectral characteristics of minerals^10–13^ and their abundance in rocks^14–16^. The few studies that have applied hyperspectral imaging to carbonate rocks in the field have focused on up-scaling and accelerating the identification of lithological heterogeneities^17,18^.

Previous studies on the reflectance of carbonate minerals have recognized up to seven absorption-bands, from 1600 to 2550 nm, caused by the vibration of the carbonate ion^10–13^. The positions, depths, and asymmetries of these bands reflect the mineral structure and properties of the substituted cations^15,19^. Calcite (Ca^+2^ mass = 40.078 amu; radius = 100 pm) has an absorption-band at ~ 2335 nm, whereas the same band for magnesite (Mg^+2^ mass = 24.305 amu; radius = 72 pm) is at ~ 2300 nm^11,12^. This absorption-band for dolomite, centered at ~ 2315 nm, is not equidistant between the calcite and magnesite band positions because the Mg–O bond is shorter than the Ca–O bond in dolomite^11^. Several studies have effectively used absorption-band positions to differentiate carbonate minerals^15,18,20,21^, but this method has not been used to discern multiple diagenetic phases in a largely monomineralic carbonate system. Furthermore, textural properties of minerals (e.g., crystal size, shape, orientation) affect the surface and volume scattering of light^11–14,22^ and can, thus, be used to further discern individual phases in carbonate rocks.

This study is based on the shortwave infrared (SWIR) hyperspectral imaging of an exposure of variably dolomitized limestone that belongs to the Cathedral Formation (Middle Cambrian; 509–497 Ma) in the Western Canadian Sedimentary Basin (WCSB). Non-stratabound dolomite bodies originate from normal-to-transtensional faults and include several diagenetic mineral phases that have distinct compositions and textures. Given that the timing and mechanism of dolomitization are well-constrained^23–26^, this succession is ideal to test whether hyperspectral imaging can be used to identify and map multiple, visibly indistinguishable, phases of dolomite in outcrop. In particular, this methodological study investigates the extent to which dolomite stoichiometry and texture can be determined by hyperspectral imaging. Consequently, the mineral distribution map products facilitate the validation and/or revision of existing fault-controlled dolomitization models^23–26^.

## Geological setting

**Figure 1:**
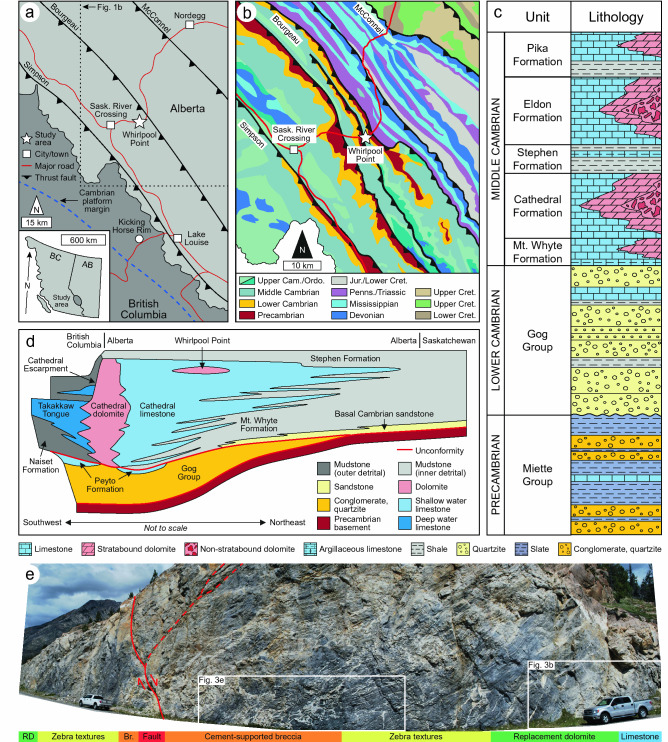
(**a**) Location of the study area in Alberta, Canada showing the major thrust faults and the Cambrian platform margin (modified from Stacey et al.^26^; based on Vandeginste et al.^37^). (**b**) Geological map of the study area (modified from Stacey et al.^26^; based on the Alberta Geological Survey Interactive Minerals Map: https://ags.aer.ca/publication/iam-001). (**c**) Stratigraphy of the southern Rocky Mountains (drafted by Dr. J. Stacey, based on Aitken^32,34^). (**d**) Schematic cross-section of the Cathedral carbonate platform in the southern Rocky Mountains (modified from Stacey et al.^26^; based on Aitken^32,34^). (**e**) Photomosaic of the Cathedral Formation at Whirlpool Point showing the diagenetic facies in relation to a normal-to-transtensional fault. Photographs provided by Dr. C. Hollis. Note that the scale changes throughout the photomosaic. Vehicle = 5 m in length.

The WCSB is a southwest-thickening wedge of sedimentary rocks, up to ~ 18 km thick in the southern Rocky Mountains, that extends from the southwest corner of the Northwest Territories to the north-central United States and includes four unconformity-bounded packages of strata^27,28^. The (1) Purcell Supergroup (Mesoproterozoic) records deposition and volcanic activity in an intracratonic basin, whereas the (2) Windermere Supergroup (Neoproterozoic) records the rifting of northwest Laurentia that waned in the Cryogenian to Ediacaran^27–29^. (3) Lower Cambrian (541–509 Ma) to Triassic strata were deposited on a passive margin. Episodic basement reactivation and renewed rifting in the Cambrian gave rise to regional thermal subsidence with evidence that heat flow and tectonic activity remained high^26,30,31^. (4) Jurassic to Paleocene strata were deposited in a foreland basin that developed during the Columbian (Jurassic to Early Cretaceous) and Laramide (Late Cretaceous to Paleocene) orogenies. The Cathedral Formation outcrops at Whirlpool Point (52°00′07.5″N, 116°28′13.5″W), the focus of this study, in the Bourgeau Thrust (Fig. 1a,b).

Middle Cambrian strata in the southern Rocky Mountains record a series of northeast-transgressing carbonate-mudrock cycles that comprise regional facies belts (Fig. 1c). The Cathedral Formation was deposited on a carbonate platform that developed with its margin proximal to the Kicking Horse Rim; an elevated paleotopographic feature that formed due to the syn-depositional reactivation of deep-rooted basement faults^32,33^. The formation is up to ~ 365 m thick and comprises a central shoal complex that grades laterally to proximal slope facies to the southwest and intrashelf basin facies to the northeast (Fig. 1d)^34,35^. At Whirlpool Point, the Cathedral Formation overlies the Mount Whyte Formation and is unconformably overlain by the Stephen Formation; host to the Burgess Shale lagerstätte^31,33^.

## Overview of the diagenetic features in the Cathedral Formation

In the southern Rocky Mountains, the Cathedral Formation consists of light-grey limestone, medium-grey to tan finely-crystalline replacement dolomite (RD), and white coarsely-crystalline saddle dolomite (SD). Dolomitization is most pervasive proximal to the Cambrian platform margin and dolomite grades laterally to limestone towards the northeast (Fig. 1d)^26^. Such dolomite bodies are typically non-stratabound (inclined-to-bedding) at their cores with stratabound (bedding-parallel) margins^24–26^. Cement-supported breccias and zebra textures are widespread in the Kicking Horse Rim area, with local occurrences of talc, magnesite, and Mississippi Valley-type (MVT) minerals^30,31,36^. These minerals are absent to the northeast, but zebra textures and cement-supported breccias are locally common^26,37^.

This study focuses on an outcrop, 240 m in width and 40 m in height, at Whirlpool Point that includes a fault-controlled dolomite body in the Cathedral Formation. The outcrop contains a fault that is oriented at 028/52, has a normal offset of 30 cm, and intersects the formation 100 m from the east end (Fig. 1e). At the core of the dolomite body, coarsely-crystalline breccias extend 25 m into the hanging-wall, 5 m into the footwall, and are limited to the fault damage zone. Proximal to the fault, the hanging-wall (146/32) comprises cement-supported breccias and bedding-inclined zebra textures. The medial part of the hanging-wall includes fabric-retentive dolomitized microbial bindstone with bedding-parallel and rare bedding-inclined zebra textures. At the margin of the dolomite body, the hanging-wall includes fabric-retentive, finely-crystalline dolomitized peloidal wackestone with rare bedding-parallel zebra textures. The lower part of the Cathedral Formation includes a sharp, bedding-parallel contact with a 2 m thick bed of limestone. In the footwall (179/25), the upper part of the formation is similar to the hanging-wall, but cement-supported breccias and bedding-inclined zebra textures are rare. The medial part of the footwall includes fabric-retentive dolomitized microbial bindstone with bedding-parallel zebra textures that grade laterally to fabric-retentive, finely-crystalline dolomite at the margin (Fig. 1e).

## Methods

### Collection and processing of the infrared reflectance data

A set of four SWIR (930–2508 nm) spectral images were acquired on June 12, 2018 between 11 am and 2 pm using a Specim SisuROCK hyperspectral scanner (a linescan imager) that is mounted on a rotary stage for wall rock imaging. Integration time varied from 5 to 10 ms depending on the time of acquisition and it required 30 s for the stage to rotate 90°. The scanner contains a 256 spectral by 320 spatial pixels mercury-cadmium-telluride detector array that acquires data at a 6.3 nm sampling interval and a 10 nm spectral bandwidth. Two Spectralon panels of 2% and 99% reflectance were positioned in each scene. Data were acquired under clear sky conditions and the sun directly illuminated the outcrop. For each scene, radiance data was obtained by applying appropriate gain and offset and conversion to reflectance, then an empirical line correction was applied based on the known reflectance of the Spectralon panels relative to their measured radiance spectrum. The latter were obtained as the mean radiance spectrum for a 20 × 20 pixel area over the panel (nominal pixel size of the acquired imagery = 5 cm). The empirical line method has the advantage of correcting for the influence of the atmosphere on the target radiance^38–40^. In contemporaneous studies with the same camera^7,41^, the wavelength position of SWIR absorptions of a National Institute of Standards and Technology referenced Mylar standard were accurate within 1 nm.

Following calibration of the spectral data to reflectance, bands with the poorest signal to noise (e.g., 930–991, 1295–1435, 1735–1998, and 2461–2508 nm) were removed from the ensuing analysis. The four images were then spatially co-registered using tie points, resulting in a single image for further analysis. Next, an iterative spatial spectral filter was used to compare the spectral similarity of spatially adjacent pixels within a 3 × 3 pixel window^42–44^. When the spectral signatures were within a predefined similarity threshold, an average spectrum was substituted for the original data, thereby reducing the intra-class spectral variability. Lastly, areas in shadow and the calibration panels were masked.

Mineralogical and lithological information in the imagery was obtained by the extraction of endmember spectra and their distributions were mapped. To derive an image endmember set, spatial-spectral endmember extraction (SSEE) was used to divide the image into equal spatial subsets (each subset = 7 × 7 pixels)^42–44^. This method is designed to discern spectrally similar endmembers that occupy different portions of the scene. The endmember set derived from SSEE was clustered and labelled to derive final endmember sets for mapping. For clustering, we used a tree cluster that recursively merges a pair of clusters based on a similarity measurement. To start, each endmember was treated as an individual cluster and endmembers that are most similar were successively merged. In this study, the Spectral Angle (SA) between two endmembers was used as the measure of similarity. A minimum SA threshold was defined to stop the merging process that took place when all pairwise clusters had a similarity greater than the threshold. To address the spectral variability of the extracted endmembers, the tree cluster tool was applied twice on the given data. The first time, using all endmembers, a SA threshold of 0.2 radians produced clusters that capture the broad material classes, namely non-geological (e.g., panels, vegetation, weathering) and geological. The next level of clustering focused on the geological class to capture subclasses and define multiple geological endmember clusters. In this case, a smaller SA threshold (0.05 radians) was used because these endmembers are more spectrally similar. Clustered endmembers were then averaged to obtain an individual endmember that represents the given class, contributing to an endmember set of thirteen geological endmembers. This clustering process was data-driven.

**Figure 2:**
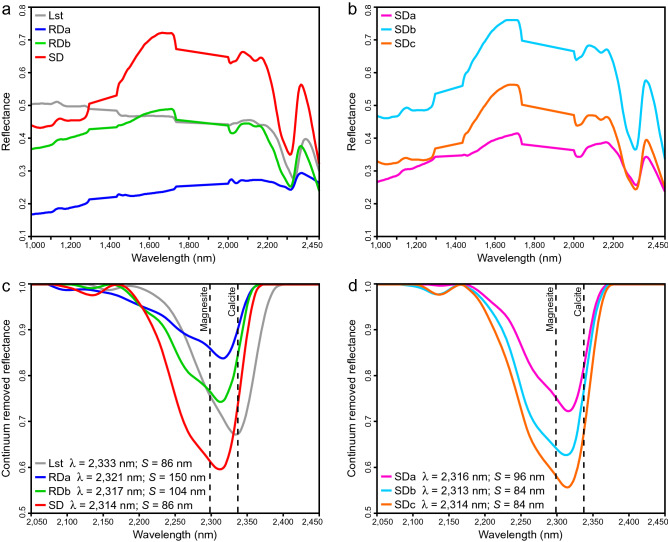
Reflectance spectra for (**a**) limestone (Lst), replacement dolomite a (RDa), replacement dolomite b (RDb), and saddle dolomite (SD). (**b**) Endmember SD consists of saddle dolomite a (SDa), saddle dolomite b (SDb), and saddle dolomite c (SDc). (**c**) Continuum removed reflectance for the ~ 2315 nm absorption-band showing Lst, RDa, RDb, and SD. (**d**) Continuum removed reflectance for the ~ 2315 nm absorption-band showing SDa, SDb, and SDc. Absorption-band positions (λ) were calculated using the linear interpolation method of van der Meer (his Fig. 2)^19^. Absorption-band asymmetry (*S*) is calculated as A–B, where A is the width from the short-wavelength shoulder to the absorption-band position and B is the width from absorption-band position to the long-wavelength shoulder^19^.

After accounting for the spectral similarity between classes, these thirteen endmembers were condensed into four groups (Fig. 2a,b): limestone (Lst), two groups of replacement dolomite (RDa, RDb), and saddle dolomite (SD). Groups were labelled based on spectral interpretations that were supported by field and petrographical observations. Group Lst was defined by all pixels with a carbonate absorption-band position > 2330 nm and was validated in the field using dilute hydrochloric acid. Group RDa includes the spectral endmembers that correspond to light- and medium- grey replacement dolomite. Endmembers that corresponded to clasts, bedding, and bedding-parallel fractures within the RDa intervals were also included. Group RDb includes the endmembers that correspond to light-brown replacement dolomite and the alteration rims along the margins of the saddle dolomite intervals. Group SD includes three subgroups (SDa, SDb, SDc), labelled based on their paragenesis, that correspond to white, coarsely crystalline, saddle dolomite. Their paragenesis was determined by the relative positions of each endmember in macro-pores that were validated by petrographical analyses.

### Distribution maps of the spectral endmembers

Mapping of the endmember spectra resulted in two image products. The first examined the distributions of limestone (Lst), replacement dolomite (RDa, RDb), and saddle dolomite (SD) and is suited for a synoptic view of the outcrop. The second, more detailed image product, examined the distribution of the saddle dolomite subgroups (SDa, SDb, SDc). In both instances, mapping the distribution of each endmember was conducted using a spectral angle mapper (SAM) algorithm that treats spectra as multidimensional vectors and computes the angle between spectral pairs^45^. For this purpose, the spectrum from each pixel of the image after processing was compared to that of each endmember. Spectra with the smallest SAM angles indicate the greatest similarity. In the first image product, the SAM results for RDa, RDb, and SD are presented as a red–green–blue composite where a higher color hue corresponds to a higher spectral similarity to the given endmember. If two or more of the endmembers predominate within a given pixel, then a color other than RGB is seen. In the second image product, the SAM results for SDa, SDb and SDc are classified in such a way that the endmember of highest similarity to that of the given pixel is assigned to that pixel. Consequently, only the colors that were assigned to the endmembers are seen in the second image product and when the SAM angle exceeds 5 degrees the pixel is not classified.

### Sampling, petrography, and geochemical analyses

Fieldwork and sampling were conducted over two field seasons. Prior to obtaining the hyperspectral reflectance data, Stacey et al.^26^ collected 72 samples from the Cathedral Formation in the Whirlpool Point area; 35 of these samples were systematically taken from the roadcut at ~ 2 m intervals along a 62 m logged section. After processing the hyperspectral data, an additional 33 samples were taken from the roadcut to support the analysis of the reflectance data and to evaluate specific features and trends within the mineral distribution maps. Samples were impregnated with blue epoxy and prepared as polished sections that were partially stained with alizarin red-S and potassium ferricyanide^46^. Polished sections were examined under plane- and cross-polarized light and then analysed using a CITL Mk5 cold cathodoluminescence (CL) system (operating conditions 15–20 kV and 350–450 μA) mounted on a Nikon Eclipse LV100N POL microscope. Dolomite crystal textures are described according to Sibley and Gregg^47^.

Thirty-eight representative samples from endmembers RDa, RDb, and SD were analysed for mineralogical composition by powder X-ray diffraction (XRD) using a Bruker D8 Advance diffractometer. Quartz was added as a standard and samples were scanned at 40 kV and 30 mA from 5° to 70° 2θ in 0.02° increments. %Ca is calculated based on Lumsden^48^ and degree of ordering is based on Goldsmith and Graf^49^. Two polished mounts that included each of the SD subgroups (SDa, SDb, SDc) were analysed by quantitative evaluation of minerals by scanning electron microscopy (QEMSCAN) and energy-dispersive X-ray (EDX) spectroscopy using an FEI Aspex eXstreme equipped with Bruker 5030 EDX detectors and an iExplorer software suite. An initial 10 × 10 mm mineral map was created for each sample at a stepping interval of 50 μm and 4 × 4 mm areas of interest were mapped at a stepping interval of 4 μm. An X-ray spectrum was generated for each point, matched against a standard library, and the map was constructed by assigning the library mineral to each point.

Twenty-four representative samples from endmembers RDa, RDb, and SD were analysed for trace elements by inductively coupled plasma mass spectrometry (ICP-MS) using an Agilent 7700× at the Advanced Isotope Geochemistry and Cosmochemistry Suite, The University of Manchester. Two polished sections that included each of the SD subgroups (SDa, SDb, SDc) were analysed by electron probe micro-analysis (EPMA) using a Cameca SX100. An initial 2 × 10 mm map was created at a stepping interval of 10 μm and 1.536 × 1.536 mm areas of interest were mapped at a stepping interval of 3 μm. Ca (Kα; PET) and Mg (Kα; TAP) were analysed at 15 kV, 10 nA, and a dwell time of 100 ms using calcite and magnesite as standards. Fe (Kα; LLIF) and Mn (Kα; LLIF) were analysed at 15 kV, 200 nA, and a dwell time of 200 ms using fayalite and tephroite as standards.

## Results

### SWIR hyperspectral imaging

Each of the endmembers can be discerned based on their absorption-band positions and spectral contrast (the difference between the peaks and the valleys in the spectrum). Phase Lst presents an absorption-band position of 2333 nm, akin to calcite and unique among all endmembers. RDa, RDb, and SD have absorption-band positions of 2321, 2317, and 2314 nm, respectively, and display increasing spectral contrast (Fig. 2c). Phase SD includes SDa, SDb, and SDc that have absorption-band positions of 2316, 2313, and 2314 nm, respectively, and display increasing spectral contrast (Fig. 2d). The absorption-band asymmetry for Lst, RDa, RDb, and SD are 86, 150, 104, and 86 nm, respectively (Fig. 2c) and the asymmetry for SDa, SDb, and SDc are 96, 84, and 84 nm, respectively (Fig. 2d).

**Figure 3:**
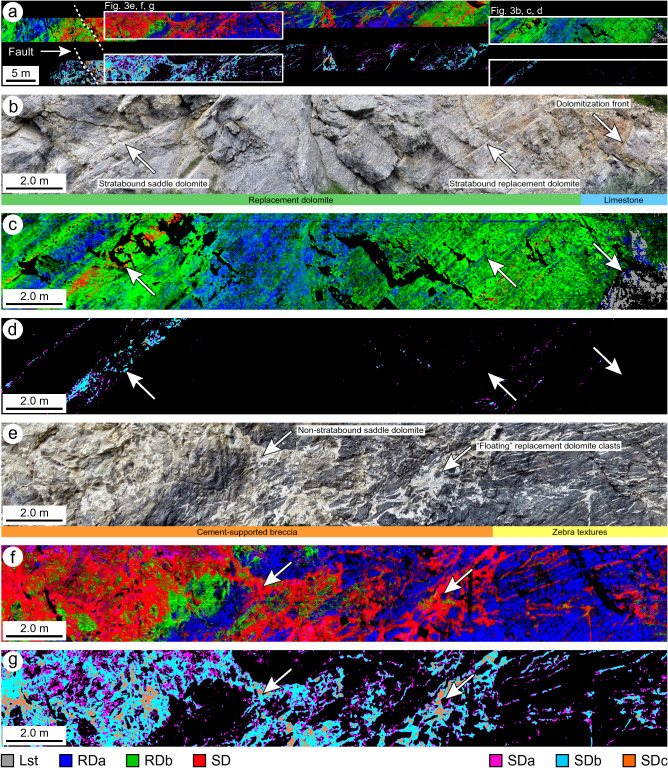
Endmember mineral distribution maps of the Cathedral Formation at Whirlpool Point showing (**a**) an overview of the acquired imagery illustrating the spatial distributions of each diagenetic phase (upper panels show Lst, RDa, RDb, and SD; lower panels show SDa, SDb, and SDc). Lst was mapped by retaining all pixels with a carbonate absorption-band position > 2330 nm. (**b**) The distal part of the fault-controlled dolomite body (white box in **a**) showing the abundant stratabound dolomite at the dolomitization front (limestone-dolomite contact) accompanied by (**c**) the corresponding Lst, RDa, RDb, and SD mineral distribution map, as well as (**d**) the SDa, SDb, and SDc mineral distribution map. (**e**) The proximal part of the fault-controlled dolomite body (white box in **a**) showing the abundant non-stratabound dolomite, cement-supported breccias, and zebra textures accompanied by (**f**) the corresponding Lst, RDa, RDb, and SD mineral map, as well as (**g**) the SDa, SDb, and SDc mineral map. Original, full-resolution, images are available in supplementary material.

At the margin of the fault-controlled dolomite body, a sharp, bedding-parallel contact occurs between Lst, RDa, and RDb (Fig. 3a, b). Scattered RDa pixels are located below the limestone-dolomite contact but RDb and SD are absent. Phase Lst is absent above this contact and throughout the remainder of the hanging-wall and the footwall. The distal parts of the hanging-wall comprise a mixture of RDa and RDb, the contacts between which follow the bedding (Fig. 3b, c). Phase SD is rare in the distal parts of the hanging-wall and is typically restricted to isolated beds of bioturbated wackestone. The spatial distribution of RDb pixels correlate with the occurrence of SD pixels, whereas RDa pixels are typically not in contact with SD pixels (Fig. 3b, c). This isolated SD bed illustrates the paragenetic sequence of each of the constituent SD phases. SDa lines the margins of the SD intervals and is post-dated by SDb. SDc is rare, but this phase is located at the centers of the SD intervals (Fig. 3b, d).

At the core of the dolomite body, the hanging-wall largely comprises cement-supported breccias and zebra textures (Fig. 3e). Consequently, phase SD is more abundant at the core of the dolomite body relative to at the margins. Isolated clasts of RDa and RDb are suspended and fully surrounded by SD (Fig. 3e, f). Typically, RDb pixels are located adjacent to SD pixels, but contacts between RDa and SD are locally common (Fig. 3e, f). The spatial distributions of the constituent phases of SD correlate with fault proximity. SDa and SDb are located throughout the hanging-wall but increase in abundance towards the core of the dolomite body (Fig. 3e, g). SDc post-dates these phases and is located at the centers of macro-pores such as breccias, fractures, zebra textures, and bedding planes. SDc increases in abundance towards the fault (Fig. 3e, g).

### Petrography

**Figure 4:**
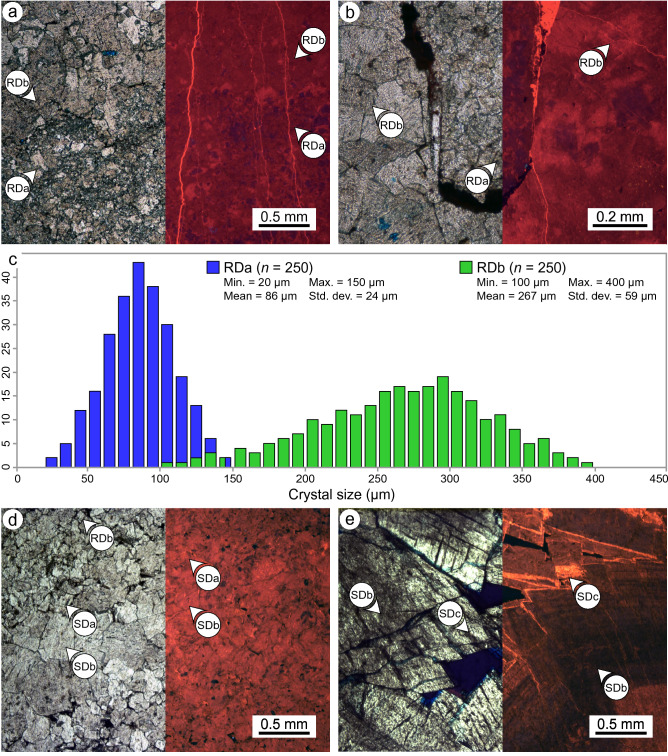
Plane polarized light (PPL; left) and cathodoluminescence (CL; right) photomicrographs of the replacement dolomite (RD) and saddle dolomite (SD) phases in the Cathedral Formation. (**a**) The contact between RDa and RDb showing their respective microscopic features. (**b**) RDa and RDb located above and below a bedding-parallel stylolite, respectively. (**c**) Crystal size histogram for RDa and RDb showing their mean crystal size, range, and standard deviation. Note that the histogram for RDb has a negative skew (− 0.3). (**d**) Bedding-parallel zebra texture showing the gradational increase in crystal size between SDa and SDb and their respective microscopic features. Note that SDa and SDb have similar CL signatures. (**e**) SDc, which commonly form rims nucleated on SDb, comprises alternating zones of dull-red to bright-red luminescent dolomite.

Petrographical analysis of samples from the Cathedral Formation identified several diagenetic phases based on their crystal size, texture, fluid inclusions, CL properties, and mineral associations (Table 1). In addition to the host limestone, two phases of RD and three phases of SD were identified. RDa includes finely-crystalline (20–150 μm), planar-e to planar-s dolomite with dull-purple luminescent cores and bright-orange to dull-red luminescent rims (Fig. 4a, b). RDb includes medium-crystalline (100–400 μm), planar-s to non-planar-a dolomite with dull-red luminescent cores and dull- to moderate-red luminescent rims (Fig. 4a, b). The crystal size distribution in RDa has a normal distribution with a mode of 84 μm, a mean of 86 μm, and a standard deviation (±) of 24 μm (Fig. 4c). The crystal size distribution in RDb is broader than RDa and has a slight negative skew (− 0.3). The modal crystal size of RDb is 284 μm with a mean of 267 μm (± 59 μm; Fig. 4c). RDa includes trace clay minerals, detrital quartz, organic matter, and pyrite that are rare in RDb. Phase SD consists of three separate phases of non-planar (saddle) dolomite that have different petrographical characteristics (Table 1).

**Table 1:** Summary table of the microscopic features of the diagenetic phases in the Cathedral Formation at Whirlpool Point, southern Rocky Mountains based on transmitted light, cathodoluminescence (CL), and scanning electron microscopy.

Diagenetic phase		Crystal size (µm)	Texture	Inclusions	CL	Other features
Matrix calcite (host limestone)	Lst	~ 25	Blocky	Turbid	Unzoned, mottled dark-purple to dull-orange	Detrital quartz, clay minerals, organic matter, and pyrite common
Replacement dolomite (RD)	RDa	20–150, mean = 86	Planar-e to planar-s	Turbid	Concentric zoning, dull-purple cores, bright-orange to dull-red rims	Fabric-retentive. Detrital quartz, clay minerals, organic matter, and pyrite locally common
	RDb	100–400, mean = 267	Planar-s to nonplanar-a	Limpid, rare inclusions	Weak blotchy zoning, dull-red cores, dull-red to moderate-red rims	Fabric-destructive. Locally associated with stylolites. Detrital quartz, clay minerals, and organic matter rare
Saddle dolomite (SD)	SDa	250–550, mean = 400	Nonplanar (saddle)	Limpid, rare inclusions	Unzoned, dull-red to medium-red	Fabric-destructive. Form syntaxial layers on RDb. Detrital quartz and clay minerals rare
	SDb	250–2500 wide, up to 4500 long, mean = 2000	Nonplanar (saddle)	Limpid	Unzoned, dull-red to medium-red	Breccia and fracture fill. Elongate crystals normal to cavity walls, crystal size increases to center
	SDc	250–1250 thick rims, crystals up to 4500, mean = 2000	Nonplanar (saddle)	Limpid	Oscillatory zoning, dull-red to bright-red, dull-orange to bright-orange	Breccia and fracture fill. Commonly form rims nucleated on SDb. Associated with Pb–Zn-Fe–Mn-Cu sulphides/oxides

SDa includes medium-crystalline (250–550 μm), non-planar dolomite that typically grew syntaxially on RDb (Fig. 4d). SDb includes coarsely-crystalline (up to 4500 μm), non-planar dolomite crystals that are abundant in cement-supported breccias, zebra textures, and fractures (Fig. 4d, e). SDb crystals are elongate, oriented normal to the cavity wall, and are polymodal in size depending on the size of the cavity in which they were precipitated. SDa and SDb are unzoned with a dull- to medium-red luminescence. SDc includes coarsely-crystalline, non-planar dolomite that are largely indistinguishable from SDb based on their textural properties. SDc crystals, however, have a characteristic dull- to bright-red and dull- to bright-orange oscillatory zonation (Fig. 4e). SDc typically form rims (250 to 1250 μm thick) that are nucleated on SDb crystals in zebra textures and fractures. Individual SDc crystals (up to 4500 μm) are common in the central parts of cement-supported breccias.

### Geochemistry

**Figure 5:**
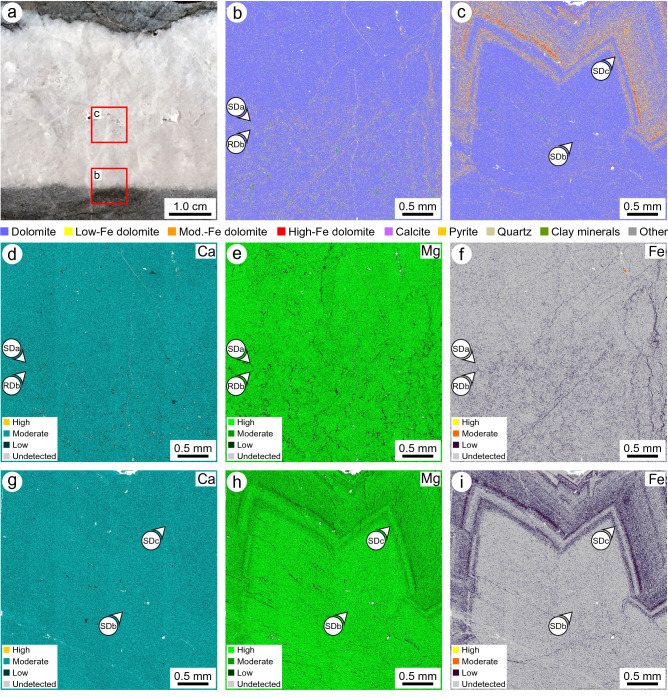
(**a**) Photograph of a sample from the Cathedral Formation showing the locations of quantitative evaluation of minerals by scanning electron microscopy (QEMSCAN) and energy-dispersive X-ray (EDX) spectroscopy images. Photographs taken by C.A. McCormick. (**b**) QEMSCAN image showing the contact between RDb and SDa. (**c**) QEMSCAN image showing the contact between SDb and SDc. (**d**, **e**, **f**) EDX spectroscopy images showing the relative abundances of Ca, Mg, and Fe across the contact between RDb and SDa. (**g**, **h**, **i**) EDX spectroscopy images showing the relative abundances of Ca, Mg, and Fe across the contact between SDb and SDc. Stepping interval of QEMSCAN and EDX spectroscopy = 4 μm.

QEMSCAN indicates that the RD phases comprise 97.87% dolomite, 1.67% clay minerals (trace illite) and muscovite, 0.41% quartz, 0.02% calcite, 0.01% pyrite, and 0.02% other minerals (Fig. 5a, b). In contrast, phase SD comprises 99.91% dolomite, 0.06% clay minerals and muscovite, 0.02% pyrite, and 0.01% calcite (Fig. 5a, c). Although RDb and SDa are clearly discernible in outcrop and hand-sample due to their color and crystallinity, EDX spectroscopy indicates that there is minimal contrast in the abundances of Ca, Mg, and Fe between these phases (Fig. 5d–f). In contrast, SDb and SDc cannot be confidently distinguished in outcrop and hand-sample, but they are clearly distinguished by their composition (Fig. 5g–i).

Each of the RD phases are stoichiometric with %Ca = 50.21 (± 0.471) and 49.79 (± 0.404) for RDa and RDb, respectively (Table 2). Phase SD has %Ca = 49.65 (± 0.540), however, the range (48.8–50.6%Ca) and standard deviation are markedly higher relative to each of the RD phases. Dolomite ordering increases from RDa (0.920 ± 0.088) to RDb (1.00 ± 0.232). The [Sr] decreases from RDa (171 ± 168 ppm) to RDb (18.1 ± 6.70 ppm). The [Fe] increases from RDa (2410 ± 2100 ppm), to RDb (4910 ± 3650 ppm), to SD (7570 ± 4270 ppm) and the [Mn] correlates with the [Fe] in each of these phases of dolomite (Table 2).

**Table 2:** Major and trace element concentrations of the diagenetic phases in the Cathedral Formation at Whirlpool Point, southern Rocky Mountains.

Phase	X-ray diffraction (XRD)	Inductively coupled plasma mass spectrometry (ICP-MS)		
	%Ca	Sr (ppm)	Fe (ppm)	Mn (ppm)
*Replacement dolomite a (RDa)*	*XRD (n* = *9)*			*ICP-MS (n* = *5)*
Min	49.27	24.7	958.0	38.6
Mean	50.21	171.3	2,407.1	182.7
Max	50.72	377.7	6,057.1	546.5
SD	0.471	167.8	2,097.6	212.7
*Replacement dolomite b (RDb)*	*XRD (n* = *12)*			*ICP-MS (n* = *13)*
Min	49.12	11.3	1444.5	101.4
Mean	49.79	18.1	4907.1	340.4
Max	50.30	37.5	11,006.0	813.3
SD	0.404	6.7	3649.7	218.3
*Saddle dolomite (SD)*	*XRD (n* = *17)*			*ICP-MS (n* = *6)*
Min	48.79	11.5	2026.1	183.6
Mean	49.65	20.2	7571.8	552.9
Max	50.57	26.8	12,290.0	813.6
SD	0.540	5.8	4272.9	278.4

Phase	Electron probe microanalysis (EPMA)			
	Ca (wt%)	Mg (wt%)	Fe (ppm)	Mn (ppm)
*Saddle dolomite a (SDa)*	*Mean %Ca* = *49.92*			
Min	15.92	8.42	0	0
Mean	21.07	12.82	2170	290
Max	26.22	16.46	4910	630
SD	2.209	1.751	1420	140
*Saddle dolomite b (SDb)*	*Mean %Ca* = *49.67*			
Min	16.13	8.85	0	0
Mean	21.06	12.94	3310	380
Max	25.99	17.03	5040	710
SD	2.202	1.744	1590	180
*Saddle dolomite c (SDc)*	*Mean %Ca* = *50.53*			
Min	14.77	7.53	0	0
Mean	20.45	12.14	12,130	1190
Max	26.13	16.75	36,740	2030
SD	2.261	1.822	2030	660

%Ca = molar Ca/(Ca + Mg).

Each of the constituent phases of SD were further analysed by EPMA (Table 2). SDa has %Ca = 49.92, SDb has %Ca = 49.67, and SDc has %Ca = 50.53. SDa has similar concentrations of Fe (2170 ± 1420 ppm) and Mn (290 ± 140 ppm) relative to SDb ([Fe] = 3310 ± 1590 ppm; [Mn] 380 ± 180 ppm). SDc is markedly enriched in Fe (12,100 ± 2030 ppm) and Mn (1190 ± 660 ppm) relative to each of the RD and the other SD phases (Table 2).

## Interpretations

### Paragenesis of the Cathedral Formation at Whirlpool Point

Petrographical observations, compositional changes, and hyperspectral imaging were used to establish the paragenesis of the Cathedral Formation. Stacey et al.^26^ documented micritized grains, post-dated by a blocky calcite cement that is consistent with cementation at or below the seafloor. Cross-cutting relationships between RDa and RDb are absent, but RDb is interpreted to have formed by the recrystallization of RDa due to (1) increasing crystal size, (2) decreasing %Ca, (3) decreasing [Sr], and (4) increasing [Fe + Mn] (Tables 1, 2). Recrystallization is associated with a change from planar-e to planar-s dolomite in RDa to planar-s to non-planar-a dolomite in RDb (Fig. 4a, b). Given that RDa and RDb are cross-cut by low-amplitude, bedding-parallel stylolites, replacement dolomitization is interpreted to have occurred during shallow burial^26,50^.

Saddle dolomite (SD) grows within pores in RDa and RDb and, therefore, post-dates them. SDa is consistently located at the margins of these pores and is overgrown and postdated by SDb (Fig. 4d). In thin-section, SDa forms a syntaxial rim that is in optical continuity with RDb; a common feature of cavity-filling cements with the same mineralogy as the cavity-wall^37^. SDa and SDb have similar compositions, gradational contacts, and were likely derived from a similar fluid-flow event. SDb crystals are an order of magnitude larger than SDa (Table 1) due to competitive crystallization; a process by which favourably-oriented crystals obstruct the growth of poorly-oriented crystals^51^. Although SDc is not present throughout the outcrop, it consistently nucleates on and, thus, postdates SDb. SDc is restricted to the central parts of vugs, fractures, zebra textures, and cement-supported breccias (Fig. 3).

### Spatial distribution of each diagenetic phase

Dolomitization fronts in the Cathedral Formation and the underlying Mount Whyte Formation at Whirlpool Point are interpreted to have “retreated” over time due to the occlusion of porosity from repeated fluid-pulses^23–26^. In this model, the core of the dolomite body is younger than the margins; with each successive fluid-pulse contributing to the recrystallization of earlier phases during the cementation of the dolomite body^23–26^. This retreating dolomitization front is associated with increased dolomite stoichiometry and ordering towards the core of the dolomite body^24^. The cement-supported breccias and associated zebra textures imaged in this study are largely restricted to the core of the dolomite body and are interpreted to have formed as a final event when the occlusion of porosity gave rise to high pore-fluid pressures and the rupturing of the formation during seismic valving^26^.

**Figure 6:**
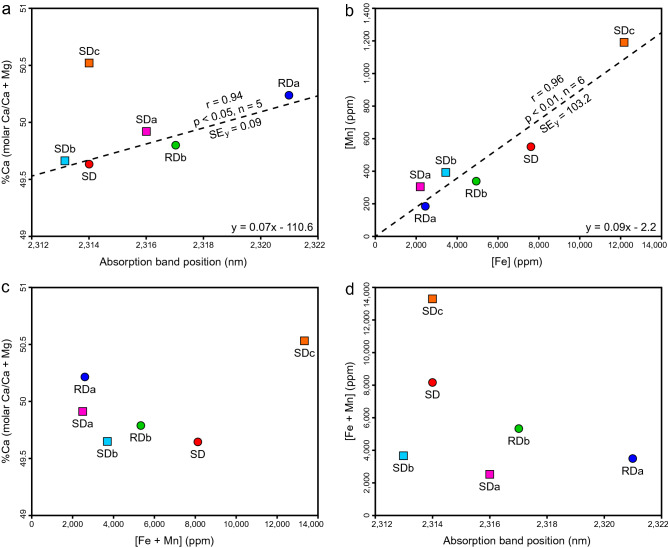
Scatterplots showing the relationships between: (**a**) %Ca versus the position of the ~ 2315 nm absorption-band, (**b**) the [Mn] versus the [Fe], (**c**) %Ca versus the [Mn + Fe], and (**d**) the [Mn + Fe] versus the position of the ~ 2315 nm absorption-band. r = correlation coefficient. SEy = standard error of the y-estimate. Note that the r and SEy for (**a**) omit SDc.

The mineral distribution maps, derived from the hyperspectral data, reveal outcrop-scale heterogeneities that provide evidence of how fault-controlled dolomitization in the Cathedral Formation progressed and terminated. Each paragenetic stage increases in abundance from the margin of the dolomite body to the core and their spectral signatures indicate that the %Ca decreases between each phase (Fig. 6). At the margin, the dolomitization front (contact between Lst and RDa) is sharp and bed-parallel (Fig. 3b, c). RDa grades laterally to RDb and the phases have a patchy distribution throughout the outcrop. Bedding planes can be identified by the distribution of RDa and RDb (Fig. 3b, c), which suggests that that they acted as permeability pathways that circulated the initial dolomitizing fluids during replacement dolomitization^52^. RDb has a spatial relationship to SD, which suggests that recrystallization occurred during the later fluid-pulses that also precipitated SD (Fig. 3). Consequently, there are textural changes from the nonstoichiometric RDa (concentrically zoned, poorly-ordered, planar-e to planar-s dolomite) at the margins of the dolomite body to the more stoichiometric RDb (weakly zoned, well-ordered, planar-s to nonplanar-a dolomite) at the core.

SD is present throughout the dolomite body, but it is more abundant at the core relative to at the margins and in the hanging-wall of the fault relative to in the footwall (Fig. 3a). Each of the constituent phases of SD, which are indistinguishable in visible light, also increase in abundance from the margin of the dolomite body to the core (Fig. 3a). SDa occurs as isolated pixels in RDa and RDb, and lines the margins of vugs, zebra textures, and cement-supported breccias (Fig. 3d, g). SDb has a gradational contact with SDa and is restricted to the central parts of these rock textures. Finally, SDc precipitated in macro-pores that were proximal to the fault (Fig. 3g), but occasionally occurs along fractures, stylolites, and bedding planes that were likely the few remaining permeability pathways after brecciation and cementation^50^. Consequently, the back-stepping and paragenesis of the SD subgroups could only be resolved using hyperspectral imaging because the endmembers are indistinguishable at the outcrop-scale and thin-sections are at too small a scale to capture their spatial distributions.

### Compositional characteristics derived from the reflectance spectra

The position of the 2315–2335 nm absorption-band is commonly used to differentiate dolomite from calcite in the laboratory^10,11^ and in remotely-sensed imagery^18,21,53^. Van der Meer^13^ suggested that this absorption-band has a linear relationship with the degree of dolomitization and Zaini et al.^14,15^ applied this approach to track the band positions of synthetic mixtures of calcite and dolomite. This methodology, however, has not been used to infer the %Ca of multiple phases of dolomite with varying compositions. RDa is the most calcium-rich phase of dolomite identified in the Cathedral Formation (%Ca = 50.21; Table 2) and has an absorption-band positioned at the longest wavelength (λ = 2321 nm; Fig. 2c). In contrast, SDb is the most magnesium-rich phase (%Ca = 49.67; Table 2), with an absorption-band positioned at the shortest wavelength (λ = 2313 nm; Fig. 2d). When SDc is excluded, the phases with intermediate compositions plot along a linear trendline with a slope of 0.07%Ca/nm and a correlation coefficient (r) of 0.94, *p* < 0.05, n = 5 (Fig. 6a).

SDc deviates from this trend because it includes substantial trace element substitution for Mg ([Fe + Mn] = 13,320 ppm; Fig. 6b, c). Increased [Fe + Mn], each with a mass greater than Ca and a radius between Mg and Ca, shift the carbonate absorption-bands to longer wavelengths^11,16^. The [Fe + Mn], however, has a poor correlation (r = 0.50, *p* > 0.05, n = 6) with the position of the ~ 2315 nm absorption-band of each of the dolomite phases in the Cathedral Formation (Fig. 6d). In the laboratory, Gaffey^11^ documented a broad Fe^+2^ absorption-band at 1200 nm and Mn^2+^ bands from 300 to 800 nm, but these features are difficult to identify in the field due to spectral range limitations of the equipment^17^. Strontium, which substitutes for Ca in dolomite^54^, reveals the recrystallization pathway from RDa (%Ca = 50.21; [Sr] = 171.3 ppm) to RDb (%Ca = 49.79; [Sr] = 18.1 ppm). The effect of [Sr] on the position of the ~ 2315 nm absorption-band, however, has not been systematically investigated in dolomite and is equivocal^12^.

Although each of the RD and SD phases are > 97% dolomite, the asymmetry of the ~ 2315 nm absorption-band generally relates to the volume of non-carbonate grains identified in thin-section. RDa (*S* = 150 nm) and RDb (*S* = 104 nm) include trace clay minerals and detrital quartz that are absent in each SD phase (*S* = 84–96 nm). QEMSCAN indicates that RD includes 1.67% clay minerals and muscovite, whereas they are negligible in SD (Fig. 5b, c). Clay minerals in RD reflect the composition of the precursor limestone, whereas their absence in SD is consistent with it being precipitated as a void-filling cement. A muscovite absorption feature at ~ 2200 nm could not be distinguished, and it is unclear if non-carbonate minerals affect the absorption-band asymmetry of each phase.

### Influence of textural characteristics on the reflectance spectra

Several laboratory-based studies have demonstrated that the textural properties of minerals and rocks impact the surface scattering and volume scattering of light^11,13,14,22^. Gaffey^11^ and Zaini et al.^14^, for example, studied the reflectance of powdered carbonate minerals that were sieved to various sizes, typically < 500 μm, and showed that finer powders have a higher overall reflectance with shallower absorption features, whereas coarser powders have a lower overall reflectance with deeper absorption features. The degree of compaction in rocks relative to powders, as well as the occlusion of porosity, can minimize these differences and it is not always possible to infer the grain or crystal size of rocks from their spectra.

In the Cathedral Formation, the endmembers present three broad groups based on their spectral contrast (Fig. 2). RDa, with a mean crystal size of 86 μm, has the lowest overall reflectance and absorption-band depths (Fig. 2a, c). Organic matter (OM), which reduces the reflectance of a sample and attenuates its absorption-bands^13^, is locally common in RDa and rare in RDb (Fig. 3a, b). Consequently, the presence of OM in RDa may contribute to its low overall reflectance. RDb and SDa (mean crystal size = 267 μm and 400 μm, respectively) have similar overall reflectance and absorption-band depths (Fig. 2). These phases are more coarsely crystalline than RDa and their spectral contrasts are intermediate between RDa and the remaining SD phases. SDb and SDc have the largest crystals (mean size = 2000 μm), roughly five times larger than SDa, and they display the highest overall reflectance and absorption-band depths (Fig. 2b, d). In this case, it is likely that the specular reflectance from crystal facets dominates over volume scattering, thus, explaining their high reflectance^55^.

## Discussion

The results of this work demonstrate that hyperspectral imaging is an invaluable tool that reveals mineralogy, composition, and texture in carbonate rocks at a macro-scale. Nevertheless, care must be taken to consider the scale of observation, or spatial resolution, because each imaged pixel comprises several phases that contribute to the reflectance^56,57^. Diagenetic phases in the Cathedral Formation, for example, comprise crystals that are below the image resolution (5 cm). Although each of the phases of dolomite are monomineralic, they are represented by an endmember because they are compositionally and texturally distinct. RDa and RDb co-exist in samples at the thin-section scale and their spectral signatures are, therefore, a linear average of their relative surface abundances. For this reason, applying the laboratory-based spectra of a single dolomite endmember to our field-based imagery would not have captured the compositional and textural diversity that is present at the outcrop-scale without petrographical and geochemical calibration. Similarly, Beckert et al.^18^ cautioned against the over-reliance on published spectral libraries, based on pure, end-member, minerals (i.e., stoichiometric well-ordered dolomite), to discriminate carbonate rocks in the field. Given that most sedimentary dolomite is nonstoichiometric with 48.0–62.5%Ca^48,58^, consists of different crystal shapes and boundaries^47^, and includes different volumes of substituted trace elements, the application of a single dolomite endmember to field- or satellite-based imagery without calibration should be treated with caution.

Initial observations of the Cathedral Formation suggests that the succession comprises only limestone, a single phase of RD, and a single phase of SD. Detailed petrographical and geochemical analyses^26^, however, revealed that the succession includes several diagenetic phases. Accordingly, this study examined the extent to which hyperspectral imaging can be used to map multiple phases of dolomite at the outcrop-scale and to determine their cross-cutting relationships; features that have traditionally only been revealed petrographically. The method presented here yields a map that illustrates the spatial distribution of each diagenetic phase in an outcrop; a feat that could not be achieved with photogrammetry or Lidar, even if supported by a dense sampling campaign^17,18^. As a result, hyperspectral imaging provides a geological toolkit that facilitates systematic sampling and improves confidence that all of the phases in the paragenetic history have been sampled, thereby allowing for macro-scale fluid flow pathways to be determined. This has economic implications because structurally-controlled deposits of magnesite and MVT-minerals (e.g., Mount Brussilof, Kicking Horse, Monarch) are associated with “hydrothermal sparry dolomite” in the Cathedral Formation^30,59,60^. Their effective exploitation, therefore, requires a robust understanding of the spatial distributions of each of the SD subgroups and their relationship to mineralization.

The methodological study presented here, with a wavelength accuracy greater than 1 nm^7,41^, tested whether hyperspectral imaging can be used to discriminate a narrow range of dolomite stoichiometries and textures in a Middle Cambrian succession. We distinguished five phases of dolomite, ranging from 49.67 to 50.21%Ca, that are approaching a stoichiometric, well-ordered endmember following 100’s of millions of years of diagenesis and several kilometers of burial^23–26^. The progressive recrystallization of dolomite over geological time is driven by mineralogical stabilization during burial that increases dolomite stoichiometry and cation ordering^61^. Consequently, this methodology has profound transferability to other, younger, successions that have not been subject to such burial, recrystallization, and mineralogical stabilization. This includes the Cenozoic “island-type” dolomite bodies that have a wider range of dolomite stoichiometries^62–64^. With careful calibration, hyperspectral imaging can provide an upscaled view of the distribution of each of these diagenetic phases in an outcrop, akin to a geocellular model, that cannot be replicated by conventional geological methods.

## Conclusions

Exposures of Middle Cambrian strata in the WCSB offer an unparalleled natural laboratory to unravel the extent and timing of dolomitization, recrystallization, and cementation in carbonate rocks. Hyperspectral imaging, in conjunction with the detailed analysis of samples from a fault-controlled dolomite body, has led to the following important conclusions:

1. The position of the ~ 2315 nm absorption-band is an effective analogue for the %Ca of dolomite and this can be used to map outcrop-scale variations in dolomite stoichiometry.
2. The relationship between %Ca and absorption-band positions is convoluted in dolomite with high trace element substitution (SDc; %Ca = 50.53; [Fe + Mn] = 13,320 ppm), therefore, non-stoichiometric dolomite can be distinguished using hyperspectral imaging.
3. The spectral contrast of the reflectance profile, which accounts for overall reflectance and absorption-band depths, correlates with the textural properties (e.g., crystal size, boundary-shape) of each dolomite phase, thus, enabling their discrimination and mapping.
4. The paragenesis and spatial distributions of the RD and SD phases in the Cathedral Formation support the prior interpretation that the dolomitization front “retreated” towards the fluid source during the ensuing recrystallization and cementation of the dolomite body.

The results of this study demonstrate that SWIR hyperspectral imaging is capable of discerning subtle diagenetic heterogeneities in carbonate rocks, beyond the routine identification of calcite and dolomite. Consequently, robust multi-scale studies can be conducted through the targeted sampling of individual diagenetic phases for further petrographical and geochemical analyses.

## Supplementary Information

Supplementary Information.

## References

[1] Cloutis EA (1996). Hyperspectral geological remote sensing: Evaluation of analytical techniques. Int. J. Remote Sens..

[2] van der Meer FD (2012). Multi-and hyperspectral geologic remote sensing: A review. Int. J. Appl. Earth Obs. Geoinf..

[3] Rivard B, Zhang J, Feng J, Sanchez-Azofeifa GA (2009). Remote predictive lithologic mapping in the Abitibi Greenstone Belt, Canada, using airborne hyperspectral imagery. Can. J. Remote. Sens..

[4] Feng J, Rogge D, Rivard B (2018). Comparison of lithological mapping results from airborne hyperspectral VNIR-SWIR, LWIR and combined data. Int. J. Appl. Earth Obs. Geoinf..

[5] Turner WA, Laamrani A, Rivard B (2003). Laboratory reflectance spectra of hydrothermally altered carbonate facies, Pine Point mining camp, NWT, Canada. Geochem. Explor. Environ. Anal..

[6] Krupnik D, Khan S (2019). Close-range, ground-based hyperspectral imaging for mining applications at various scales: Review and case studies. Earth Sci. Rev..

[7] Lypaczewski P (2019). Using hyperspectral imaging to vector towards mineralization at the Canadian Malartic gold deposit, Québec, Canada. Ore Geol. Rev..

[8] Bellante GJ, Powell SL, Lawrence RL, Repasky KS, Dougher TAO (2013). Aerial detection of a simulated CO_2_ leak from a geologic sequestration site using hyperspectral imagery. Int. J. Greenh. Gas Control.

[9] Zabcic N, Rivard B, Ong C, Müller A (2014). Using airborne hyperspectral data to characterize the surface pH and mineralogy of pyrite mine tailings. Int. J. Appl. Earth Obs. Geoinf..

[10] Hunt GR, Salisbury JW (1971). Visible and near-infrared spectra of minerals and rocks: II. Carbonates. Mod. Geol..

[11] Gaffey SJ (1986). Spectral reflectance of carbonate minerals in the visible and near infrared (0.35–2.55 microns): calcite, aragonite, and dolomite. Am. Mineral..

[12] Gaffey SJ (1987). Spectral reflectance of carbonate minerals in the visible and near infrared (0.35–2.55 μm): Anhydrous carbonate minerals. J. Geophys. Res. Solid Earth.

[13] van der Meer F (1995). Spectral reflectance of carbonate mineral mixtures and bidirectional reflectance theory: Quantitative analysis techniques for application in remote sensing. Remote Sens. Rev..

[14] Zaini N, van der Meer F, van der Werff H (2012). Effect of grain size and mineral mixing on carbonate absorption features in the SWIR and TIR wavelength regions. Remote Sens..

[15] Zaini N, van der Meer F, van der Werff H (2014). Determination of carbonate rock chemistry using laboratory-based hyperspectral imagery. Remote Sens..

[16] Green D, Schodlok M (2016). Characterisation of carbonate minerals from hyperspectral TIR scanning using features at 14,000 and 11,300 nm. Aust. J. Earth Sci..

[17] Kurz TH (2012). Hyperspectral image analysis of different carbonate lithologies (limestone, karst and hydrothermal dolomites): the Pozalagua Quarry case study (Cantabria, North-west Spain). Sedimentology.

[18] Beckert J, Vandeginste V, McKean TJ, Alroichdi A, John CM (2018). Ground-based hyperspectral imaging as a tool to identify different carbonate phases in natural cliffs. Int. J. Remote Sens..

[19] van der Meer F (2004). Analysis of spectral absorption features in hyperspectral imagery. Int. J. Appl. Earth Obs. Geoinf..

[20] Windeler DS, Lyon RJP (1991). Discriminating dolomitization of marble in the Ludwig Skarn near Yerington, Nevada using high-resolution airborne infrared imagery. Photogramm. Eng. Remote Sens..

[21] van der Meer F (1996). Classification of remotely-sensed imagery using an indicator kriging approach: Application to the problem of calcite-dolomite mineral mapping. Int. J. Remote Sens..

[22] Crowley JK (1986). Visible and near-infrared spectra of carbonate rocks: Reflectance variations related to petrographic texture and impurities. J. Geophys. Res. Solid Earth.

[23] Koeshidayatullah A (2020). Evaluating new fault-controlled hydrothermal dolomitisation models: Insights from the Cambrian Dolomite Western Canadian Sedimentary Basin. Sedimentology.

[24] Koeshidayatullah A (2020). Origin and evolution of fault-controlled hydrothermal dolomitization fronts: A new insight. Earth Planet. Sci. Lett..

[25] Koeshidayatullah, A., Corlett, H., & Hollis, C. An overview of structurally-controlled dolostone-limestone transitions in the stratigraphic record. *Earth Sci. Rev.* 103751 (2021).

[26] Stacey J (2021). Regional fault-controlled shallow dolomitization of the Middle Cambrian Cathedral Formation by hydrothermal fluids fluxed through a basal clastic aquifer. Geol. Soc. Am. Bull..

[27] Bond GC, Kominz MA (1984). Construction of tectonic subsidence curves for the early Paleozoic miogeocline, southern Canadian Rocky Mountains: Implications for subsidence mechanisms, age of breakup, and crustal thinning. Geol. Soc. Am. Bull..

[28] Desjardins PR, Buatois LA, Pratt BR, Mángano MG (2010). Stratigraphy and sedimentary environments of the Lower Cambrian Gog Group in the southern Rocky Mountains of Western Canada: Transgressive sandstones on a broad continental margin. Bull. Can. Pet. Geol..

[29] Li ZX (2008). Assembly, configuration, and break-up history of Rodinia: A synthesis. Precambr. Res..

[30] Powell WG, Johnston PA, Collom CJ, Johnston KJ (2006). Middle Cambrian brine seeps on the Kicking Horse Rim and their relationship to talc and magnesite mineralization and associated dolomitization, British Columbia, Canada. Econ. Geol..

[31] Johnston PA, Johnston KJ, Collom CJ, Powell WG, Pollock RJ (2009). Palaeontology and depositional environments of ancient brine seeps in the Middle Cambrian Burgess Shale at The Monarch, British Columbia, Canada. Palaeogeogr. Palaeoclimatol. Palaeoecol..

[32] Aitken JD (1971). Control of lower Paleozoic sedimentary facies by the Kicking Horse Rim, southern Rocky Mountains, Canada. Bull. Can. Pet. Geol..

[33] Collom CJ, Johnston PA, Powell WG (2009). Reinterpretation of ‘Middle’ Cambrian stratigraphy of the rifted western Laurentian margin: Burgess Shale Formation and contiguous units (Sauk II megasequence), Rocky Mountains, Canada. Palaeogeogr. Palaeoclimatol. Palaeoecol..

[34] Aitken JD (1997). Stratigraphy of the Middle Cambrian platformal succession, southern Rocky Mountains. Geol. Surv. Can. Bull..

[35] Pratt BR (2002). Teepees in peritidal carbonates: Origin via earthquake-induced deformation, with example from the Middle Cambrian of western Canada. Sed. Geol..

[36] Vandeginste V (2007). Geochemical constraints on the origin of the Kicking Horse and Monarch Mississippi Valley-type lead-zinc ore deposits, southeast British Columbia, Canada. Miner. Depos..

[37] Vandeginste V (2005). Zebra dolomitization as a result of focused fluid flow in the Rocky Mountains Fold and Thrust Belt, Canada. Sedimentology.

[38] Kurz TH, Buckley SJ, Howell JA, Schneider D (2011). Integration of panoramic hyperspectral imaging with terrestrial lidar data. Photogram. Rec..

[39] Kurz TH, Buckley SJ, Howell JA (2013). Close-range hyperspectral imaging for geological field studies: Workflow and methods. Int. J. Remote Sens..

[40] Murphy RJ, Taylor Z, Schneider S, Nieto J (2015). Mapping clay minerals in an open-pit mine using hyperspectral and LiDAR data. Eur. J. Remote Sens..

[41] Lypaczewski P, Rivard B (2018). Estimating the Mg# and Al^VI^ content of biotite and chlorite from shortwave infrared reflectance spectroscopy: Predictive equations and recommendations for their use. Int. J. Appl. Earth Obs. Geoinf..

[42] Rogge DM (2007). Integration of spatial–spectral information for the improved extraction of endmembers. Remote Sens. Environ..

[43] Rogge, D.M. & Rivard, B. Iterative spatial filtering for reducing intra-class spectral variability and noise. In *IEEE GRSS workshop on hyperspectral image and signal processing: Evolution in remote sensing*, Reykjavik, Iceland, June 14–16, 1–4 (2010).

[44] Rogge D (2012). Spatial sub-sampling using local endmembers for adapting OSP and SSEE for large-scale hyperspectral surveys. IEEE J. Sel. Top. Appl. Earth Observ. Remote Sens..

[45] Kruse FA (1993). The spectral image processing system (SIPS)–interactive visualization and analysis of imaging spectrometer data. Remote Sens. Environ..

[46] Dickson JAD (1965). A modified staining technique for carbonates in thin section. Nature.

[47] Sibley DF, Gregg JM (1987). Classification of dolomite rock textures. J. Sediment. Res..

[48] Lumsden DN (1979). Discrepancy between thin-section and X-ray estimates of dolomite in limestone. J. Sediment. Res..

[49] Goldsmith JR, Graf DL (1958). Structural and compositional variations in some natural dolomites. J. Geol..

[50] Martín-Martín JD (2018). Activation of stylolites as conduits for overpressured fluid flow in dolomitized platform carbonates. Geol. Soc. Lond. Spec. Publ..

[51] Wallace MW, Hood AvS (2018). Zebra textures in carbonate rocks: fractures produced by the force of crystallization during mineral replacement. Sediment. Geol..

[52] Budd DA, Park AJ (2018). Formation of bed-scale spatial patterns in dolomite abundance during early dolomitization: Part I. Mechanisms and feedbacks revealed by reaction transport modelling. Sedimentology.

[53] Beirami MR, Tangestani MH (2020). A new band ratio approach for discriminating calcite and dolomite by ASTER imagery in arid and semiarid regions. Nat. Resour. Res..

[54] Vahrenkamp VC, Swart PK (1990). New distribution coefficient for the incorporation of strontium into dolomite and its implications for the formation of ancient dolomites. Geology.

[55] van Ginneken B, Stavridi M, Koenderink JJ (1998). Diffuse and specular reflectance from rough surfaces. Appl. Opt..

[56] Woodcock CE, Strahler AH (1987). The factor of scale in remote sensing. Remote Sens. Environ..

[57] van der Meer F (2012). Remote-sensing image analysis and geostatistics. Int. J. Remote Sens..

[58] Jones B, Luth RW, MacNeil AJ (2001). Powder X-ray diffraction analysis of homogeneous and heterogeneous sedimentary dolostones. J. Sediment. Res..

[59] Paradis, S. & Simandl, G. J. Is there a genetic link between the SEDEX and MVT deposits of the Canadian Cordillera? In Rogers, N. (Ed.). *Targeted Geoscience Initiative: 2016 Report of Activities*, Geological Survey of Canada, Open File 8199, 107–113 (2017). 10.4095/299573.

[60] Paradis, S. & Simandl, G. J. Are there genetic links between carbonate-hosted barite-zinc-lead sulphide deposits and magnesite mineralization in southeast British Columbia? In: Rogers, N. (Ed.). *Targeted Geoscience Initiative: 2017 Report of Activities*, Geological Survey of Canada, Open File 8358, 217–227 (2017). 10.4095/306391.

[61] Manche CJ, Kaczmarek SE (2021). A global study of dolomite stoichiometry and cation ordering through the Phanerozoic. J. Sediment. Res..

[62] Budd DA (1997). Cenozoic dolomites of carbonate islands: Their attributes and origin. Earth Sci. Rev..

[63] Ren M, Jones B (2018). Genesis of island dolostones. Sedimentology.

[64] Wang R (2021). Dolomitization micro-conditions constraint on dolomite stoichiometry: A case study from the Miocene Huangliu Formation, Xisha Islands. South China Sea. Mar. Pet. Geol..

