# Supplementary material for: Shortwave infrared hyperspectral imaging as a novel method to elucidate multi-phase dolomitization, recrystallization, and cementation in carbonate sedimentary rocks
Source: Sci Rep. 2021 Nov 5;11:21732. doi: 10.1038/s41598-021-01118-4 (PMC8571368; doi:10.1038/s41598-021-01118-4)
Supplement: Supplementary file 1 — Supplementary Information. [file 41598_2021_1118_MOESM1_ESM.pdf]

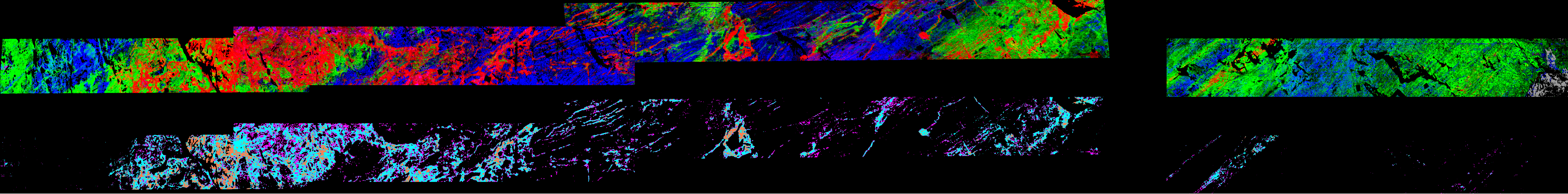

Supplementary material 1. (a) Endmember mineral distribution maps of the Cathedral Formation at Whirlpool Point showing limestone (Lst.; grey), replacement dolomite a (RDa; blue), replacement dolomite b (RDb; green), and saddle dolomite (SD; red). (b) Endmember mineral distribution maps of the Cathedral Formation at Whirlpool Point showing saddle dolomite a (SDa; pink), saddle dolomite b (SDb; aqua), and saddle dolomite c (SDc; orange).
